# Colorectal Cancer Related to Chronic *Strongyloides stercoralis* Infection

**DOI:** 10.1155/2020/8886460

**Published:** 2020-09-07

**Authors:** M. Sava, T. Huynh, A. Frugoli, L. Kong, M. Salehpour, B. Barrows

**Affiliations:** ^1^Western University of Health Sciences, Ventura, CA, USA; ^2^Community Memorial Health Systems, Graduate Medical Education, Ventura, CA, USA; ^3^Community Memorial Health Systems, Graduate Medical Education, Department of Internal Medicine, Ventura, CA, USA; ^4^Community Memorial Health Systems, Graduate Medical Educations, Department of Hematology Oncology, Ventura, CA, USA; ^5^Community Memorial Hospital, Department of General Surgery, Ventura, CA, USA; ^6^Community Memorial Hospital, Department of Pathology, Ventura, CA, USA

## Abstract

The majority of individuals with *Strongyloides stercoralis* (*S. stercoralis*) colitis are clinically asymptomatic. Symptomatic individuals may complain of nonspecific gastrointestinal symptoms, such as abdominal pain, intermittent or persistent diarrhea, nausea, or loss of appetite. In addition, skin manifestations such as recurrent urticaria can occur. If infection is not diagnosed and left untreated, it can lead to chronic inflammation of the gastrointestinal tract. It is well documented that chronic colitis such as inflammatory bowel disease can predispose individuals to colorectal cancer. Additionally, there is evidence of chronic parasitic infections inducing the development of cancers in other organs within the gastrointestinal tract. In this case vignette, we describe a case of chronic *Strongyloides stercoralis* infection in a Peruvian woman presenting with colorectal cancer.

## 1. Introduction

Strongyloidiasis is caused by *S. stercoralis*, a soil-transmitted helminth, endemic in tropical and subtropical regions including Latin America, sub-Saharan Africa, and Southeast Asia [1–3]. Worldwide, between 30 and 100 million people are infected. However, in the United States, a series of small studies in select populations have shown that between 0% and 6.1% of persons sampled were infected by *S. stercoralis* [4]. The presence of infection is greater for immigrants to the United States. The total prevalence of *S. stercoralis* is likely underestimated because of the low sensitivity of testing, nonspecific symptoms, and no clear guidelines for testing in nonendemic areas. *S. stercoralis* infection can persist for decades in a single patient due to its autoinfection life cycle. Infectious agents, including parasites, often have oncogenic potential. According to Parkin et al., the global health burden of infection associated with cancer in 2002 included 17.8% of the malignancy burden. The infections associated with malignancy range from chronic bacterial infections, such as *Helicobacter pylori*, viral etiologies including viral hepatitis, and, in rare cases, flukes or parasites can also contribute to cancer development [5]. Multiple studies have reported that *S. stercoralis* may be a cofactor in HTLV-1-related T-cell lymphoma in endemic areas [6, 7]. In addition, a number of case reports have described the occurrence of gastrointestinal cancer with *S. stercoralis* infection, suggesting that the parasite may be a risk factor for gastrointestinal malignancy [3, 5, 8, 9]. Contrary to this, some hypothesize that gastrointestinal malignancy is a risk for infection.

This case vignette describes a rare parasitic infection with review of microbe-associated malignancy literature and suggests possible hypotheses for an association between chronic *S. stercoralis* infection and colorectal cancer.

## 2. Case Report

A 70-year-old Peruvian female with a history of gastroesophageal reflux disease, osteoporosis, and hyperlipidemia was admitted for worsening abdominal pain and nonbloody diarrhea with associated weight loss and early satiety. The patient first noticed an increase in her vague abdominal pain with diarrhea after she returned from a trip to Peru about six weeks prior. She frequently travels to Peru to visit family at least biannually. She reported chronic intermittent nonspecific symptoms of abdominal pain and bloating for years. Upon return from her recent trip, she was prescribed sulfamethoxazole and trimethoprim for a total of three days for possible traveler's diarrhea and her symptoms subsided. The following week, she developed upper abdominal pain that gradually became more severe. It was worse with direct pressure and relieved with rest. She also had unintentional weight loss of five pounds in the last month. Her last colonoscopy was done in 2012 (nearly 7 years prior, in Peru), which was unremarkable except for diverticulosis without polyps. She has no family history of colon polyps, colorectal cancer, celiac disease, or inflammatory bowel disease (IBD).

Physical exam was notable for subumbilical and right lower quadrant tenderness. A complete blood count with differential revealed normocytic anemia (hemoglobin 10.9 g/dL) and peripheral eosinophilia (13.1%). Laboratory studies revealed iron deficiency, albumin 3.4 g/dL, and CEA 2.1. A stool PCR panel was positive for enteroaggregative *Escherichia coli* (EAEC) and enteropathogenic *Escherichia coli* (EPEC). Stool ova and parasite (O&P) examination was positive for *S. stercoralis*, and a fecal occult blood test was also positive. Review of medical records revealed new-onset anemia for the last year and intermittent eosinophilia (19%) for the last 3 years, with multiple health care visits for gastrointestinal complaints.

**Figure 1:**
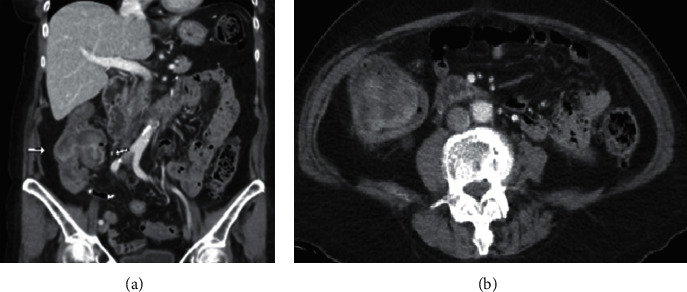
Right ascending mass in ascending colon CT. (a) (Arrows) colo-colonic intussusception with associated submucosal edema. (b) Right colonic mass measuring approximately 7 cm.

**Figure 2:**
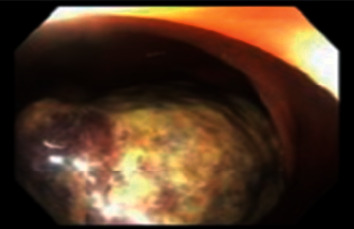
Images from colonoscopy demonstrating pedunculated large mass in the cecum.

The patient underwent computed tomography (CT) of the abdomen and pelvis with intravenous contrast, which demonstrated marked irregularity of the right ascending colon and sigmoid with heterogeneous enhancing right colonic mass (∼7 cm) with a few small adjacent lymph nodes (Figure 1). There was colo-colonic intussusception with associated submucosal edema. She underwent follow-up colonoscopy that demonstrated a nearly obstructing pedunculated mass with malignant appearance in the right ascending colon near the cecum (Figure 2), a 10 mm sessile polyp in the descending colon, and moderate diverticulosis of the left colon. The colonic mass was biopsied in multiple areas, and the polyp excised. Biopsy of the mass revealed invasive adenocarcinoma, and the descending colon polyp was found to be a tubulovillous adenoma.

**Figure 3:**
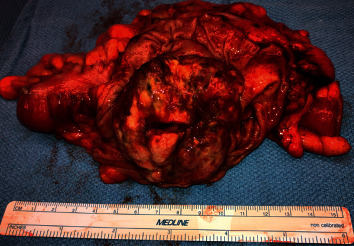
Gross pathologic specimen demonstrating a large tumor.

**Figure 4:**
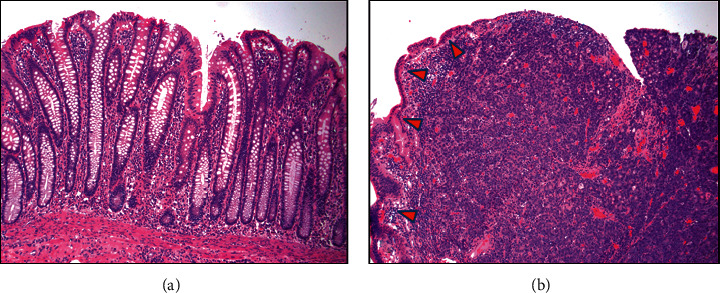
(a) Uninvolved colon from the colectomy specimen with minimal reactive changes and no significant acute or chronic inflammation (100× magnification). (b) Colon mass from the colectomy specimen showing poorly differentiated invasive adenocarcinoma with superficial ulceration, sheet-like architecture, and minimal gland formation undermining adjacent reactive colonic epithelium (arrowheads).

Given the patient's stool exam was positive for *S. stercoralis* with gastrointestinal symptoms, the patient was treated with ivermectin 200 *μ*g/kg on day one and repeated 24 hours later, with an additional dose at two weeks. Due to the size of the obstructing mass with risk of colon perforation, the patient underwent exploratory laparotomy with right hemicolectomy and reanastomosis (Figure 3). Pathologic evaluation of the specimen revealed stage IIIB with poorly differentiated adenocarcinoma with lymphovascular invasion, perineural invasion, and 3 of 38 lymph nodes involved. Sections of the uninvolved colon were unremarkable and showed no inflammation or other features suggestive of *S. stercoralis* infection (Figure 4(a)). Histologic examination of the mass showed sheet-like architecture of the malignant cells with scattered foci of gland formation, consistent with poorly differentiated adenocarcinoma (Figure 4(b)). Sections of the carcinoma also demonstrated numerous tumor infiltrating lymphocytes and scattered lymphocyte aggregates at the periphery of the tumor consistent with a Crohn's-like inflammatory response. Mismatch repair protein immunohistochemistry for possible hereditary nonpolyposis colon cancer (HNPCC) showed absent nuclear expression of MLH1 and PMS2. However, the MLH1 methylation study was positive for hypermethylation (59.4%; ref <20%), and a BRAF V600E mutation was identified by molecular studies, consistent with sporadic adenocarcinoma (not associated with HNPCC). Up to 15% of colon cancers show altered expression of mismatch repair proteins correlating with microsatellite instability in tumor cells. However, the majority of these cases are due to sporadic loss of MLH1 expression as a result of somatic/epigenetic inactivation of MLH1 (promoter hypermethylation). The presence of BRAF V600E mutation is helpful since this mutation has been found in sporadic microsatellite unstable tumors, but not in HNPCC-associated cancers [10]. Risks and benefits of oral chemotherapy with capecitabine and oxaliplatin and IV chemotherapy with oxaliplatin were discussed, including adjuvant therapy.

## 3. Discussion

*S. stercoralis* infection in humans starts with infectious filariform larvae penetrating the skin, entering venous circulation, and migrating to the lungs [4]. The organisms are then carried to the pharynx and swallowed [4]. This cycle can be accelerated by ingestion of contaminated water containing filariform larvae. In the small intestine, the larvae develop into adult females, which reproduce asexually and release eggs into the gastrointestinal tract [4, 11]. The persistence of *S. stercoralis* infection is due to the parasite's unusual capability to undergo an autoinfective cycle in absence of reinfection [12]. Autoinfection is characterized by the ability of rhabditiform larvae in the large intestine to transform into infectious filariform larvae, then penetrate the intestinal mucosa, and migrate to other organs including the lungs, restarting the cycle [4]. Another peculiarity of *S. stercoralis* is its capacity to cause hyperinfection syndrome, as a result of uncontrolled overproliferation of filariform larvae in stool and sputum, leading to the clinical manifestation of larval migration with development or exacerbation of gastrointestinal and pulmonary symptoms [3]. Hyperinfection syndrome primarily occurs in immunocompromised individuals and can have a high mortality rate (approaching 100%) if not diagnosed and treated [13]. Patients with *S. stercoralis* infection may have a variety of symptoms involving the gastrointestinal tract (abdominal pain and diarrhea), skin (itching and urticaria), and respiratory tract (asthma-like symptoms and dyspnea) [4]. In a meta-analysis, urticaria showed a strong association with *S. stercoralis* infection, while diarrhea and abdominal pain had a weaker association [12].

The gold standard for the diagnosis of strongyloidiasis is serial stool examination. Routine stool examination identifies up to 30% of cases because of low infectious burden and intermittent larval excretion. Thus, repeat stool collection of multiple samples can increase the sensitivity of the test to 80–90%. Eosinophilia is an important marker in nematode parasite infections and is common with strongyloidiasis. The eosinophil count may be normal in patients with severe strongyloidiasis or immunosuppression [11]. The patient in our case report demonstrated absolute eosinophilia (up to 1000/*μ*L; ref 0–500/*μ*L) and a positive stool O&P examination, which aided in making the diagnosis, so appropriate treatment could be started. We suggest that the parasitic load was high enough to stimulate the immune system to increase production of eosinophils in this patient.

The connection between infection and malignancy is well studied. Knoll et al. in their expert review reported that “infections are estimated to be responsible for up to 25% to 50% of all cancers that occur in humans.” In their 18 Pearl review, they provide examples of infections caused by viruses, bacteria, parasites, and fungi with their contribution to the development of malignancy. A summary of the prior research investigating infection-associated malignancy risk states infections can promote carcinogenesis by three main mechanisms: chronic inflammation due to the persistence of an infectious agent in the host; insertion of oncogenes into the host genome; and reduced immune surveillance as a result of immunosuppression [14]. Two of the most well-known flukes or parasites associated with carcinogenesis include *Schistosoma haematobium* infection and its association with cancer of the urinary bladder; and the liver flukes, *Clonorchis sinensis* and *Opisthorchis viverrini*, which are associated with cholangiocarcinoma of the liver [15]. Despite the classification of these specific parasites as carcinogens, no correlation has been proposed for *S. stercoralis* in colorectal cancer. *S. stercoralis* colitis often remains asymptomatic for decades, predisposing individuals to chronic inflammation of the gut and colon. Mucosal damage caused by parasites, their eggs, or secreted products can lead to restorative hyperplasia of damaged tissue. Additionally, *S. stercoralis*, like other parasites, could have immunomodulating capabilities that alter the immune response between Th1 and Th2 signaling, which would provide a microenvironment that hinders immune surveillance [16]. This immune modulation has been well documented with other parasites including protozoa such as *Toxoplasma gondii* and *Trypanosoma cruzi* as outlined by Callejas et al. [15]. Physical damage and/or immunomodulation may create a microenvironment conducive to the development of neoplasia [5]. A large cohort study of 5209 cancer patients investigated the association of coinfection with *S. stercoralis* and HTLV-1 with cancer and showed that *S. stercoralis* infection was associated with an increased occurrence of liver cancer and lymphoma [7]. A proposed mechanism for this interaction suggests the *S. stercoralis* antigen may stimulate HTLV-1-infected cell replication by activating the IL-2/IL-2R system in dually infected patients, potentially fostering the development of adult T-cell leukemia in this population [17]. A case report describing a Korean patient presenting with both *S. stercoralis* infection and early gastric adenocarcinoma showed *S. stercoralis* adults and eggs associated with gastric adenocarcinoma and adenoma tissues suggesting a possible causative role of *S. stercoralis* infection [8]. Another case report identified *S. stercoralis* in a duodenal biopsy from a patient found to have colorectal cancer, which also suggests a potential correlation between *S. stercoralis* and cancer development [3]. An association between colorectal cancer with chronic *S. stercoralis* has also been reported in a Columbian patient case report [18]. Hirata et al. demonstrated an increased prevalence of *S. stercoralis* infection in patients with biliary tract cancer compared to control patients, suggesting *S. stercoralis* infection as a risk factor for malignancies in the hepato-pancreato-biliary system [9]. The associations between parasitic infections and human cancers are well documented. Two parasites, *S. haematobium and O. viverrini*, are definitive causes of cancer while *S. mansoni, S. japonicum,* and *Clonorchis sinensis* are well known to be associated with cancer. However, studies demonstrating the correlation between *S. stercoralis* infection and cancer are rare in the literature.

In our case, we were able to detect *S. stercoralis* larvae in the patient's stool samples; however, we were unable to detect eggs or worms in the patient's surgical biopsy or colorectal adenocarcinoma (after the first dose of ivermectin was administered). Review of outpatient records did demonstrate chronic GI complaints. Our observations and the aforementioned studies suggest that *S. stercoralis* infection, leading to chronic inflammation, may not only serve as a cofactor for induction of HTLV-1 lymphoma but may also stimulate induction of colon carcinogenesis by direct epithelial injury and/or activation of the host immune response. However, additional research is needed to identify specific mechanisms concerning the role of *S. stercoralis* infection in colon carcinogenesis [15]. An alternative argument includes that patients with immunosuppression are at higher risk of developing parasitic infection. The study conducted by Machado et al. addresses the alternative hypothesis by looking at the frequency of *S. stercoralis* infection in patients with gastrointestinal cancer versus other types of cancer (bladder, breast, melanoma, lung, and uterus). The study shows that the diagnosis of *S. stercoralis* infection was significantly higher in patients with gastrointestinal cancer than in non-gastrointestinal cancer patients [19]. In another study evaluating the prevalence of *S. stercoralis* infection in patients being treated for cancer, it was shown that the overall prevalence of *S. stercoralis* infection in these patients was low [6].

## 4. Conclusion

*Strongyloides stercoralis* infection is among the most neglected and elusive parasitic conditions worldwide. Our case report is among the few cases that support an association between gastrointestinal cancer and chronic *S. stercoralis* infection. We propose that chronic *S. stercoralis* infection may be a risk factor for gastrointestinal cancer. Further research to clarify the mechanism of increased cancer risk is needed to emphasize the importance of ruling out *S. stercoralis* infection in cancer patients of endemic areas, particularly those with gastrointestinal malignancy.

## Data Availability

No data were used to support this study.
